# Intravenous versus oral paracetamol in a UK ambulance service: a case control study

**DOI:** 10.29045/14784726.2020.06.5.1.1

**Published:** 2020-06-01

**Authors:** Karl Charlton, Matthew Limmer, Hayley Moore

**Affiliations:** North East Ambulance Service NHS Foundation Trust: ORCID iD: https://orcid.org/0000-0002-9601-1083; North East Ambulance Service NHS Foundation Trust; North East Ambulance Service NHS Foundation Trust

**Keywords:** analgesia, emergency medical services, pain management

## Abstract

**Objectives:**

To determine the effectiveness of intravenous versus oral paracetamol (acetaminophen) in the management of acute pain in the out-of-hospital setting.

**Methods:**

We extracted ambulance electronic patient care records for all patients who received 1 g intravenous paracetamol throughout January 2019, and case matched these by sex and age with consecutive patients who received 1 g oral paracetamol over the same time period. Eligible for inclusion were all patients aged ≥ 18 who received 1 g paracetamol for acute pain and who were transported to the emergency department (ED). The primary outcome was the mean reduction in pain score using the numeric rating scale (NRS), with a reduction of 2 or more accepted as clinically significant.

**Results:**

80 care records were eligible for analysis; 40 patients received intravenous and 40 patients received oral paracetamol. The mean age of both groups was 54 years (± 3 years) and 67.5% (*n* = 54) were female. Patients receiving intravenous paracetamol had a clinically significant mean (SD) improved pain score compared to those receiving oral paracetamol, 2.02 (1.64) versus 0.75 (1.76), respectively [p = 0.0013]. 13/40 (32.5%) patients who received intravenous paracetamol saw an improved pain score of ≥ 2 compared to 8/40 (20%) who received oral paracetamol. No patients received additional analgesia or reported any adverse symptoms. Abdominal pain, infection and trauma were the most common causes of pain in both groups.

**Conclusion:**

Our study suggests that intravenous paracetamol is more effective than oral paracetamol when managing acute pain in the out-of-hospital setting. Our findings support further investigation of the role of paracetamol in paramedic practice using more robust methods.

## Introduction

Paracetamol (acetaminophen) is one of the most commonly used non-opioid analgesic agents, used in various healthcare settings because of its efficacy (Duggan & Scott, 2009; Prescott, 1996) and favourable adverse reaction profile (Prescott, 2000).

Paracetamol presents in several formulations, including for oral and intravenous administration, although it remains unclear if intravenous paracetamol is more effective than oral administration for patients in acute pain (Jibril et al., 2015).

Efficient pain management is one of the most important elements of emergency care (Jalili et al., 2016). Pain is a common symptom for those who present to the ambulance service, with 20% reporting moderate to severe pain (McLean et al., 2004). Various analgesic options exist to manage pain in the out-of-hospital setting, including oral and intravenous paracetamol, non-steroidal anti-inflammatories (NSAIDs) and opiate-based medicines (Berben et al., 2011; Mura et al., 2017). However, use of these analgesic options is considered inadequate (Dißmann et al., 2018).

National ambulance service clinical practice guidelines categorise pain as mild (score 1–3), moderate (score 4–6) and severe (score 7–10) (JRCALC, 2019). Paramedics routinely score pain using a numeric rating scale (NRS), where a respondent verbalises a whole number (0–10) that best reflects their pain (Hawker et al., 2011). Intravenous morphine is the standard analgesia used by paramedics to control moderate to severe pain (Carr et al., 2019) despite its recognised often deleterious effects (Dixon et al., 2018) and increased length of hospital stay (Stephan et al., 2010). A paucity of high quality evidence exists upon which to base out-of-hospital analgesic treatment, and paramedics are encouraged to use their clinical judgement to inform decisions regarding therapeutic interventions, often leading to variability and suboptimal pain management (McManus & Sallee, 2005).

## Objectives

Intravenous paracetamol was introduced to the North East Ambulance Service (NEAS) NHS Foundation Trust’s medicine formulary in 2018. We set out to determine if intravenous paracetamol is more effective than oral paracetamol to manage acute pain in the out-of-hospital setting measured using the NRS. Our null hypothesis accepted equivalence of both formulations.

Findings are reported in accordance with the Strengthening the Reporting of Observational Studies in Epidemiology (STROBE) statement (Von Elm et al., 2007).

## Methods

### Study design

We set out to collect data from patients who received 1 g intravenous or oral paracetamol between 1 January and 31 January 2019 inclusive. We extracted ambulance electronic patient care records for all patients who received 1 g intravenous paracetamol, and case-matched these care records with consecutive patients who received 1 g oral paracetamol over the same time period. We first matched each case by sex and then by age. We excluded care records from both groups that only recorded one pain score or those where the patient required additional analgesia. Data were related to patients attended to by an NEAS paramedic, and include patients from across the North East of England, from both a rural and urban population. Eligible participants met the criteria in Table 1.

**Table 1.** Eligibility criteria.

Inclusion criteria	Exclusion criteria
Adult patients aged ≥ 18 years of age	Incomplete care records
Received oral or IV paracetamol for acute pain	
Transported to ED	

In addition to demographic data, we collected patient information relating to the aetiology of pain and first and last numeric pain scores. The primary outcome was the mean improvement in pain in each group, measured using the NRS, with a reduction of 2 or more points recognised as clinically significant (Farrar et al., 2001) and accepted as a measure of clinical significance for this study.

### Data analysis

Data were analysed within their respective groups to facilitate comparative analysis. Data were analysed using MedCalc version 19.0.5. All data were normally distributed. Differences between means were evaluated by an independent samples t-test. Differences in proportions were evaluated using a comparison of proportions test. For all cases, a p value of < 0.05 was considered statistically significant.

## Results

In total, 80 care records were eligible for analysis; 40 eligible patients received 1 g intravenous paracetamol and these were matched with 40 consecutive eligible patients who received 1 g oral paracetamol. All patients in both groups were given a single 1 g dose of paracetamol by a paramedic to manage acute pain. The mean age of each group was 54 years and each intravenous care record was matched with an oral care record, first by sex and then age (± 3 years). Patients in both groups were more likely to be female (Table 2).

**Table 2.** Age and sex of included patients stratified by route of paracetamol administration.

Route of administration	Intravenous	Oral
No. patients	40	40
Mean age in years (age range)	54.22 (22–95)	54.52 (22–94)
Sex n (%)	27 (67.5)	27 (67.5)

A statistically significant number of patients in our sample reported moderate to severe pain, 65/80 (81%), p < 0.0001. Patients who received intravenous paracetamol had a higher mean first pain score than patients receiving oral paracetamol, 7.17 (± 5) versus 5.37 (± 4). These patients also had a clinically significant improvement in mean pain score, 2.02, SD 1.64, p < 0.0001 versus 0.75, SD 1.76, p = 0.1593. The difference in improved pain score between patients receiving intravenous versus oral paracetamol was 1.21 and was statistically significant, p = 0.0013 (Table 3). 13/40 (32.5%) patients who received intravenous paracetamol saw an improved pain score of ≥ 2 compared to 8/40 (20%) who received oral paracetamol.

**Table 3.** Summary of results.

Route of paracetamol administration	Intravenous	Oral	p-value
**Pain scores**			
Mean first pain score (SD)	7.17 (2.07)	5.37 (2.41)	0.0006
Mean last pain score (SD)	5.15 (2.00)	4.62 (2.31)	
Mean difference between first and last pain score (SD)	2.02 (1.64)	0.75 (1.76)	0.0013
95% CI of mean pain score difference	1.11–2.93	0.30–1.80	
**Presumed aetiology of pain n (%)**			
Abdominal pain	21 (52.5)	6 (15)	0.0004
Acute coronary syndrome	0	3 (7.5)	0.0794
Gynaecological	0	2 (5.0)	0.1547
Neurological pain	1 (2.5)	3 (7.5)	0.3079
Infection	6 (15)	12 (30)	0.1104
Musculoskeletal	2 (5.0)	2 (5.0)	1.0000
Trauma	10 (25)	12 (30)	0.6187

Paracetamol was administered in both formulations for a variety of causes of acute pain, with abdominal and traumatic aetiologies being the most common. The causes of pain did not differ significantly between both groups, except for abdominal pain where intravenous rather than oral paracetamol was the formulation of choice, 21/40 (52.5%) compared to 6/40 (15%), p = 0.0004 (Table 3).

There were no reported adverse events in either group. No patients who received intravenous paracetamol reported a worsening pain score compared to one patient who received oral paracetamol.

## Discussion

Currently, there is limited research evaluating intravenous versus oral paracetamol for patients presenting in acute pain, with most research focusing on postoperative analgesia. Our study set out to explore the use of paracetamol in the out-of-hospital setting and to improve our understanding of the use of analgesia in emergency care. Given that 81% (65/80) of the patients in our sample reported moderate to severe pain, there is a great need for high quality evidence to underpin practice.

To our knowledge, this is the first study to compare intravenous versus oral paracetamol in the out-of-hospital setting. Our results found that 1 g intravenous paracetamol is more effective to manage acute pain than 1 g oral paracetamol. Some of the patients in our study were advised to take their own oral paracetamol when they made the initial emergency call but none had taken any other analgesia. This reflects normal clinical practice, as it would be unethical to leave patients in pain unnecessarily. The patients in our study were similar in that each patient who received intravenous paracetamol was closely matched to a patient who received oral paracetamol, by sex and age. The mean first pain score of each group differed significantly.

The causes of pain varied across both groups but, except for abdominal pain, this was insignificant. The patients in our study contacted the ambulance service at all times of day and night.

Our research contrasts greatly with findings by Furyk et al. (2017), whose research into intravenous versus oral paracetamol in the ED arguably influences current paramedic guidelines. Their study found that intravenous paracetamol was equivalent to oral paracetamol, despite patients in the intravenous group reporting consistently lower pain scores at all time points. In their randomised controlled trial, all patients received intravenous opiates prior to randomisation and consequently had some degree of pain control at baseline. We argue that these patients differ greatly from those in our study and are not reflective of the wider population who present to the ambulance service in pain. In addition, Furyk et al. (2017) excluded patients who attended the ED at night, when pain is known often to be more intense, exacerbated by other factors, and to require a wide range of analgesic options to control (Hart et al., 1970).

Oral paracetamol does come with several advantages; it is easy to administer, low risk and is familiar with patients and paramedics alike, which in the out-of-hospital setting is of significant benefit.

Some argue that intravenous paracetamol is more effective than its oral counterpart due to how the intravenous formulation is absorbed by the body. In their study, Brett et al. (2012) measured post-operative plasma paracetamol levels in patients randomised to receive intravenous or oral paracetamol. Plasma concentration levels are known to reflect therapeutic thresholds and determine analgesic effect. All patients randomised to the intravenous group had plasma levels above the analgesic level compared to less than half in the oral group. This may explain why in our study pain was manged more effectively in the intravenous paracetamol group. Brett et al. (2012) also identified a trend towards a reduction in the requirement for rescue medications and a shortened length of stay, which we were unable to corroborate in our work.

These findings echo research by Hansen et al. (2018), who investigated the use of intravenous versus oral paracetamol in hysterectomy patients. In their research, patients who received intravenous paracetamol as an adjunct to opioid analgesia had a shortened length of stay, lower hospitalisation costs and lower daily opioid dependency, compared to those who received oral paracetamol.

While we did not compare the use of intravenous paracetamol and opioid analgesia, several studies report a reduction in opioid usage when intravenous paracetamol is given compared to when oral formulations are used (Bollinger et al., 2015; Hansen et al., 2018). Given the well-documented adverse effects and increased healthcare costs associated with the use of opioid analgesia, there may be benefits in encouraging paramedics to use intravenous paracetamol rather than morphine as a first-line analgesia, although further work is required to underpin this change in practice. No patients in our study reported adverse effects from the use of either formulations of paracetamol.

The NRS is a unidimensional, single-item scale used to measure pain intensity, and it reflects current clinical paramedic practice. The scale is simple to use and is easily understood by various population groups. Furthermore, it is associated with high test-retest reliability (Hawker et al., 2011) and is recommended for use in prehospital pain measurement (Maio et al., 2002). The NRS is not without limitation, however, in that it is unable to accommodate patients who cannot verbally score their pain and it fails to capture the full complexity of pain.

It is clearly not appropriate or necessary for all patients experiencing pain in the out-of-hospital setting to be offered intravenous paracetamol. There are risks that preclude justification of recommending the routine use of intravenous paracetamol, such as local infection and phlebitis (Furyk et al., 2017). Intravenous paracetamol should be given with caution in those with a known risk of hepatotoxicity, and adjusted doses are required for those of low body weight (Macario & Royal, 2011). In addition, there are increased costs associated with the use of intravenous paracetamol, with each 1 g intravenous dose costing approximately £1.50 compared to 4 p for the oral equivalent at the time of writing this article (NHS Business Services Authority, 2019). This poses significant implications for ambulance services when deciding how best to support the use of intravenous paracetamol.

### Strengths and limitations

We have matched each case in our sample with two controls (age and sex) and, except for abdominal pain, the aetiology of pain is similar across both groups, enhancing the external validity of our findings. However, the observational design of our study leaves our results subject to bias and confounding. Patients receiving intravenous paracetamol were more likely to report moderate to severe pain compared to those who received the oral equivalent, who were more likely to report mild to moderate pain. Consequently, we would expect to see a greater improvement in those patients who received intravenous paracetamol. There was variability in how long patients had experienced pain and when pain was measured by the attending paramedic. Consequently, we were unable to measure pain control at the same time points for each patient. Paramedics were not blinded to treatment allocation and we were unable to account for paramedic preference of either formulation; consequently, our findings may be subject to observer bias.

Our study is not statistically powered, although we have analysed data for 80 patients which is a comparable sample size to several similar studies, albeit of different design.

Although all patients in our study were transported to ED, we were unable to link our findings to hospital data so we cannot correlate an improved mean out-of-hospital pain score with an improved experience in hospital, or with wider benefits to the healthcare system such as reduced length of stay.

Pain is subjective, and a mean reduction in pain score is not a reflection of patient satisfaction. Some patients may still have been in considerable pain despite a significant improvement in their NRS.

## Conclusions

Our study suggests that intravenous paracetamol is more effective than oral paracetamol when managing acute pain in the out-of-hospital setting. Several factors such as cost and appropriateness may preclude the routine use of intravenous paracetamol, however. Our research lacks the validity and robustness of a randomised controlled trial, and further opportunities exist to evaluate intravenous versus oral paracetamol in the out-of-hospital setting.

### Implications for practice

To our knowledge, this is the first study to evaluate the role of intravenous versus oral paracetamol in the out-of-hospital setting. Our study highlights that intravenous paracetamol is more effective than oral paracetamol in managing pain in the acute phase of emergency care. Paramedics should consider the use of intravenous paracetamol more readily, particularly when patients report a high pain score.

## Author contributions

KC designed the study, carried out data collection and wrote the article. ML designed the study, and commented on and provided critical revision of the article. KC, ML and HM carried out data analysis. KC acts as the guarantor for this article.

## Conflict of interest

None declared.

## Ethics

Favourable ethical opinion was sought and received from Newcastle and North Tyneside 1 research ethics committee 19/NE/0197 and the health research authority.

## Funding

None.
